# Sex differences in utilisation of extracorporeal membrane oxygenation support and outcomes in Taiwan

**DOI:** 10.1186/s12871-023-02045-9

**Published:** 2023-03-20

**Authors:** Feng-Cheng Chang, An-Hsun Chou, Yu-Tung Huang, Jhen-Ling Huang, Victor Chien-Chia Wu, Chih-Hsiang Chang, Kuo-Chun Hung, Shang-Hung Chang, Pao-Hsien Chu, Shao-Wei Chen

**Affiliations:** 1 Department of Anesthesiology, Chang Gung Memorial Hospital, Linkou Medical Center, Chang Gung University, Taoyuan City, Taiwan; 2 Center for Big Data Analytics and Statistics, Chang Gung Memorial Hospital, Linkou Medical Center, Taoyuan City, Taiwan; 3 Department of Cardiology, Chang Gung Memorial Hospital, Linkou Medical Center, Taoyuan City, Taiwan; 4 Division of Nephrology, Department of Internal Medicine, Linkou Chang Gung Memorial Hospital, Taoyuan City, Taiwan; 5 Division of Thoracic and Cardiovascular Surgery, Department of Surgery, Chang Gung Memorial Hospital, Linkou Medical Center, Chang Gung University, No. 5, Fuxing St., Guishan Dist., Taoyuan City, 33305 Taiwan

**Keywords:** Extracorporeal membrane oxygenation, ECMO, Sex differences, Mortality, Women

## Abstract

**Background:**

The impact of sex-related differences in patients receiving extracorporeal membrane oxygenation support (ECMO) support is still inconclusive. This population-based study aimed to investigate sex differences in short- or long-term outcomes in order to improve clinical practice.

**Methods:**

Patients who received ECMO between 2001 to 2017 were identified from the Taiwan National Health Insurance Research Database. Propensity score matching with a 1:1 ratio was conducted in female-to-male groups, to reduce confounding of baseline covariates. Outcomes included in-hospital mortality, all-cause mortality, all-cause readmission, and ECMO-related complications. Logistic regression analysis, Cox proportional hazard model, and join point regression were used to compare sex differences in both short- or long-term outcomes.

**Results:**

In total, 7,010 matched patients from 11,734 ECMO receivers were included for analysis. The use of ECMO increased dramatically in past years, although the proportion of females was still lower than males. There was a decreasing trend of females undergoing ECMO over time. Female patients have lower risks of in-hospital mortality (64.08% in females vs 66.48% in males; *P* = 0.0352) and ECMO-related complications compared with males. Furthermore, females also had favorable long-term late outcomes such as all-cause mortality (73.35% in females vs 76.98% in males; *P* = 0.009) and readmission rate (6.99% in females vs 9.19% in males; *P* = 0.001).

**Conclusions:**

Female patients had more favorable in-hospital and long-term survival outcomes. Despite improvement in modern ECMO technique and equipment, ECMO remains underutilized in eligible female patients. Thus, females should undergo ECMO treatment if available and indicated.

**Trial registration:**

The institutional review board of Chang Gung Memorial Hospital approved all data usage and the study protocol (registration number: 202100151B0C502; date of registration: 23/08/2021).

**Supplementary Information:**

The online version contains supplementary material available at 10.1186/s12871-023-02045-9.

## Background

Extracorporeal membrane oxygenation (ECMO) support, as one technique of mechanical circulatory support, provides cardiac and respiratory support for critically ill patients with various indications. ECMO is widely used in numerous scenes, including the intensive care unit, emergency department, and even the operating room as extracorporeal life support [1]. In addition to traditional indications such as post-infarction cardiogenic shock [2] and respiratory failure [3]. ECMO has also been adopted for postcardiotomy support [4], major trauma [5], bridge to heart–lung transplantation [6], and extracorporeal cardiopulmonary resuscitation [7].

Over the past decades, the number of patients receiving ECMO support has increased exponentially worldwide as a result of improvements in technique and extended indications [6, 8]. Although several previous articles have documented the clinical outcomes of ECMO use [8–10], there have been negligible discussion focused on sex differences, much less within a large population-based cohort study. Therefore, gaps remain in our knowledge regarding sex-related differences. The aim of the present study was to demonstrate the effects of sex differences on clinical outcomes of critically ill patients receiving ECMO by using information obtained from a population-based claims database in Taiwan.

## Methods

### Data source

This population-based cohort study was implemented based on data obtained from the Taiwan National Health Insurance Research Database (NHIRD). The NHIRD was derived from the government-operated single-payer National Health Insurance (NHI) program. The NHI scheme covers 99.8% of the population in Taiwan and has reimbursed medical expenditures of ECMO since 2002.

The NHIRD contains data on all registered beneficiaries, including all outpatient, inpatient, and pharmacy-dispensing claims from medical providers contracted with the NHI administration. We collected details of critically ill patients receiving ECMO, such as indications, demographic distribution, and in-hospital, and/or long-term follow-up outcomes. All data in the NHIRD are encrypted and de-identified, but the data remain linkable and limited to research purposes only [11]. Moreover, we linked these patients to the encrypted Death Registry data for follow-up of all-cause mortality. Both the NHIRD research committee and the institutional review board of Chang Gung Memorial Hospital approved all data usage and the study protocol and waived the need for individual informed consent (registration number: 202100151B0C502).

### Study population

**Fig. 1:**
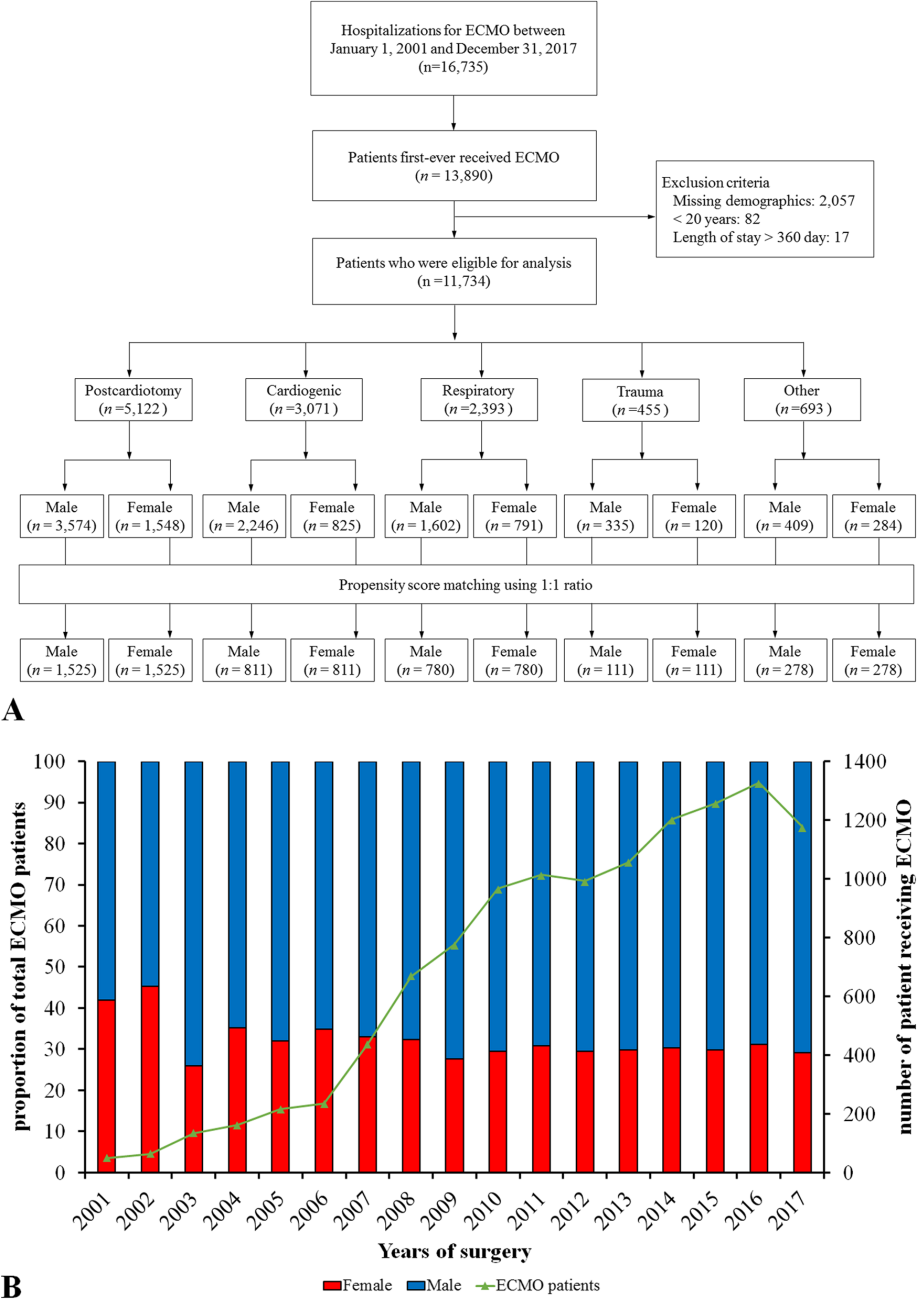
Flow chart of patient inclusion and exclusion (**A**) and trend of patients receiving ECMO support among both sexes (**B**) across the years. ECMO, extracorporeal membrane oxygenation support

By examining the NHI reimbursement codes, we identified hospitalization records of all patients receiving ECMO intervention between January 1, 2001, and December 31, 2017. We excluded patients with missing demographic detail, age less than 20 years, and length of stay longer than 360 days. After applying the exclusion criteria, 11,734 patients were eligible for analysis. These patients were subsequently categorized into five groups (postcardiotomy, cardiogenic, respiratory, trauma, and all others) by indications, and all groups were further stratified into male and female patients (Fig. 1A). To reduce the confounding of baseline covariates for comparison, we applied the propensity score matching (PSM) method with a 1:1 ratio in each of the five categorized groups in the female and male groups. Finally, a total of 7,010 patients were included in the matched cohort.

### Comorbidities

We collected information on demographic and clinical characteristics, including age, sex, monthly income, urbanization, year of surgery and hospital level where the patients received ECMO. We used the *International Classification of Diseases, 9th Revision, Clinical Modification* (ICD-9-CM), and *10th Revision* (ICD-10-CM) diagnostic codes to detect comorbid diseases. Comorbidities were identified via any inpatient diagnosis before the index ECMO intervention. Medication prescription history was determined using the NHI reimbursement codes in the 6 months before the index hospitalization. We also identified the indications of ECMO support by ICD-9-CM and ICD-10-CM procedure codes (Supplemental Table 1).

### Outcomes

We analyzed both short-term outcomes and late follow-up outcomes using the ICD-9-CM, ICD-10-CM, and NHI reimbursement codes. The outcomes of primary interest in this study were in-hospital mortality and long-term survival including overall survival and mortality after discharge. Secondary outcomes included in-hospital complications and follow-up comorbidities. Each patient was followed from the index ECMO intervention to the date of death, the date of outcome occurrence, or December 31, 2017, whichever came earlier.

### Subgroup analysis

In addition to the analysis of entire cohort, we also performed the subgroup analysis to demonstrated the sex differences for in-hospital and follow-up outcomes as described above for patients with different indications of the 4 groups (postcardiotomy, cardiogenic, respiratory, and trauma).

### Statistical analysis

We used the absolute standardized mean difference to determine substantial differences in both the female and male groups before and after PSM. An absolute value of the standardized difference of < 0.1 was considered a negligible difference. To reduce the confounding of different variables, we adopted the PSM method to eliminate the selection bias. The covariates used in the matching were listed in Supplemental Table 2, including age, monthly income, level of urbanization, year of surgery, comorbidities, level of hospitals, cumulative volume of ECMO across study period, preoperative medications, and ECMO indications. The propensity score was the predicted probability of being in the female group given the covariate values using multivariable logistic regression. We used a greedy nearest neighbor algorithm with a caliper of 0.20 times the standard deviation of the logit of the propensity score to process the matching, with random matching order and without replacement. The PSM with a 1:1 ratio was conducted in the female-to-male groups.

We adopted univariate logistic regression and linear regression to test the in-hospital outcomes, including in-hospital mortality, associated complications, and other in-hospital parameters. To compare the risk of all-cause mortality and other fatal time to event outcomes, such as all-cause mortality after discharge and cardiovascular death, we performed survival analyses, including Kaplan–Meier and the Cox proportional hazard model. Moreover, we used the Fine and Gray subdistribution hazard model to analyze the incidences of nonfatal time to event outcomes, such as revascularization and acute myocardial infarction, while considering all-cause mortality to be competing risk.

We used univariate logistic regression analysis to test the trend of proportion of female patients receiving ECMO support across the study years. In addition, to identify changes in in-hospital mortality rate trends, we estimated join point regression for the female, male, and overall groups using the Joinpoint Regression Program, version 4.5.0.1 (Statistical Research and Applications Branch, National Cancer Institute). A two-sided *P* value < 0.05 was considered statistically significant. Statistical analyses were performed using SAS version 9.4 (SAS Institute, Cary, NC).

## Results

### Baseline characteristics

Supplemental Table 2 summarizes and compares the demographics and clinical and surgical characteristics between sexes. There was no significant sex-related difference in age, socioeconomic status, hospital level where patients received ECMO, or duration of support. There was a tendency for female patients to have fewer comorbid conditions, such as prior myocardial infarction and chronic obstructive pulmonary disease; however, females suffered from depression more often than males did. Males were more likely to have a prescription for anti-platelets and alpha-blockers than females were, although we found no other remarkable difference in medication history. With regard to the indications for ECMO support, females tended to have a respiratory cause, whereas males had more cardiogenic indications. To ensure the distribution of matching variables was well-balanced between the groups, we performed PSM, and the absolute standardized difference of each variable was < 0.1. In addition, all sex differences in patient baseline characteristics were negligible after PSM (Supplemental Table 2).

### Epidemiology of ECMO use and outcome in Taiwan

**Fig. 2:**
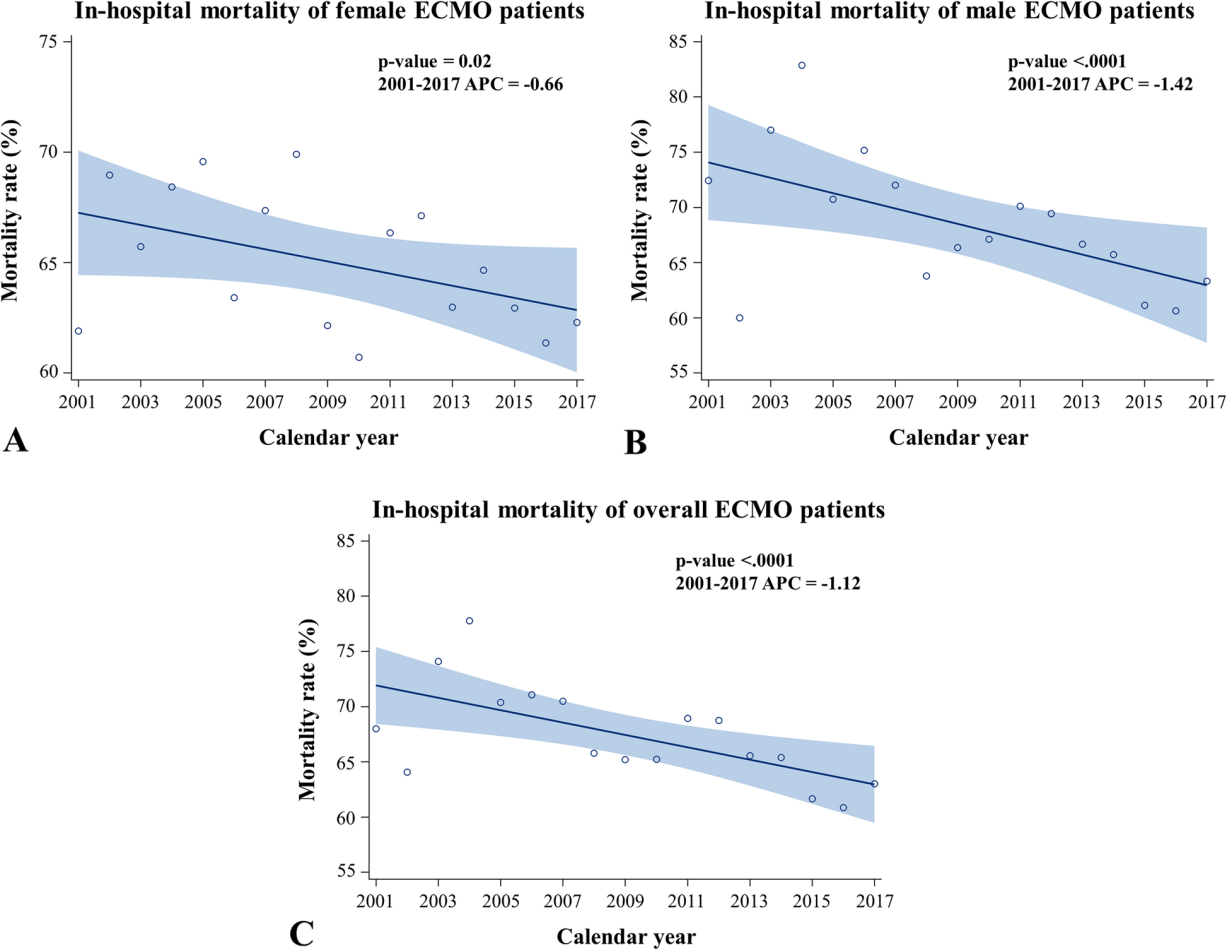
Trending of in-hospital mortality of patients receiving ECMO support: female (**A**), male (**B**), and overall (**C**) populations. ECMO, extracorporeal membrane oxygenation support

Figure 1B shows that the number of patients who received ECMO support increased dramatically from 2001 to 2017. There was also a decreasing trend of females undergoing ECMO (*P* = 0.0524) across the time course. With regard to the trend of in-hospital mortality, as shown in Fig. 2, there was a gradual decrease in in-hospital mortality across the years in female (*P* = 0.02; Fig. 2A), male (*P* < 0.001; Fig. 2B), and overall (*P* < 0.001; Fig. 2C) patients, respectively. Various mortality rates were provided by age group, including in-hospital mortality, all-cause mortality during follow-up, mortality after discharge and CV death after discharge (Supplemental Tables 3 to 6). In general, these various types of mortality increased with advanced age in both females and males. Besides, females had overall lower mortality compared to males across most of the age groups, especially in the younger age groups.

### In-hospital mortality and ECMO-related complications

Our findings show that the real-world overall in-hospital mortality rates of patients receiving ECMO support were 64.18% and 66.09% for female and male patients, respectively. Moreover, the in-hospital mortality rate after PSM was significantly different between the sexes, with a rate of 64.08% in the female group and 66.48% in the male group (odds ratio [OR], 0.90; 95% confidence interval [CI], 0.82–0.99; *P* = 0.0352). In terms of complications, female patients had a lower risk of new-onset dialysis (OR, 0.83; 95% CI, 0.76–0.91; *P* < 0.001), prolonged ventilation, and deep wound infection after ECMO (OR, 0.85; 95% CI, 0.76–0.95; *P* = 0.005) as compared with males. Nevertheless, a higher risk of new-onset hemorrhagic stroke in females was noted (OR, 1.71; 95% CI, 1.20–2.44; *P* = 0.003). We also demonstrated the lower in-hospital cost in female patients compared to males ($28.24 × 10^3^ versus $30.30 × 10^3^; *P* = 0.001). Other results including the amount of blood transfusion and overall hospital stay days were not significantly different between the sexes (Table 1).

**Table 1:** In-hospital outcomes and ECMO-related complications in the female and male groups

Variable	Female (*n* = 3505)	Male (*n* = 3505)	OR/B (95% CI)	*P value*
In-hospital mortality	2246 (64.08)	2330 (66.48)	0.90 (0.82–0.99)	0.035
New-onset stroke	168 (4.79)	156 (4.45)	1.08 (0.87–1.35)	0.495
New-onset ischemic stroke	103 (2.94)	113 (3.22)	0.91 (0.69–1.19)	0.490
New-onset hemorrhagic stroke	83 (2.37)	49 (1.4)	1.71 (1.20–2.44)	0.003
Fasciotomy or amputation	57 (1.63)	68 (1.94)	0.84 (0.59–1.19)	0.323
Prolonged ventilation (> = 7 days)	1763 (50.31)	1877 (53.56)	0.88 (0.80–0.97)	0.008
Newly-onset dialysis	1537 (43.85)	1698 (48.45)	0.83 (0.76–0.91)	<0 .001
Deep wound infection after ECMO	775 (22.11)	875 (24.96)	0.85 (0.76–0.95)	0.005
PRBC amount (U)	11.61 ± 11.12	11.56 ± 11.32	0.05 (-0.50–0.59)	0.863
FFP amount (U)	10.96 ± 12.02	11.13 ± 11.90	-0.17 (-0.83–0.49)	0.613
Platelet amount (U)	8.44 ± 10.92	8.57 ± 11.15	-0.13 (-0.73–0.48)	0.677
Ventilator (days)	10.06 ± 9.75	10.88 ± 10.33	-0.82 (-1.31– -0.34)	0.001
ICU duration (days)	11.06 ± 9.57	11.55 ± 10.09	-0.49 (-0.97– -0.02)	0.041
Hospital stays (days)	26.53 ± 32.26	27.99 ± 33.59	-1.47 (-3.04–0.10)	0.067
In-hospital cost (USD × 10^3^)	28.24 ± 23.19	30.30 ± 26.49	-2.06 (-3.24– -0.87)	0.001

Values are given as number (%) or mean ± standard deviation

*ECMO* Extracorporeal membrane oxygenation, *OR* Odds ratio, *B* unstandardized coefficient, *PRBC* Packed red blood cell, *FFP* Fresh frozen plasma, *ICU* Intensive care unit, *USD* United States dollar

### Follow-up late outcomes

Table 2 presents the late outcomes and shows that females had a lower risk of all-cause mortality during follow-up. The all-cause mortality rates after PSM were 73.35% in the female group and 76.98% in the male group (OR, 0.93; 95% CI, 0.88–0.98; *P* = 0.009). In addition, the outcomes of cardiovascular death (*P* < 0.0001), all-cause readmission (*P* = 0.001), admission for heart failure (*P* = 0.02), and revascularization (*P* = 0.001) were also favored in female patients. However, there was no significant in acute myocardial infarction, ischemic stroke, hemorrhagic stroke, or end-stage renal disease requiring permanent dialysis between the sexes.

**Table 2:** Long-term outcomes in the female and male groups

Variable	Female	Male	HR or SHR	*P value*
	(*n* = 3505)	(*n* = 3505)	(95% CI)	
All-cause mortality	2571 (73.35)	2698 (76.98)	0.93 (0.88–0.98)	0.009
All-cause mortality after discharge	325 (25.81)	368 (31.32)	0.74 (0.64—0.87)	< .001
CV death	106 (8.42)	161 (13.7)	0.56 (0.44–0.71)	< .0001
Revascularization (PCI or CABG)	8 (0.64)	28 (2.38)	0.26 (0.12–0.56)	0.001
Acute myocardial infarction	26 (2.07)	36 (3.06)	0.64 (0.39–1.07)	0.088
Ischemic stroke	21 (1.67)	27 (2.3)	0.67 (0.38–1.19)	0.174
Hemorrhagic stroke	12 (0.95)	21 (1.79)	0.51 (0.25–1.03)	0.061
All-cause of readmission	767 (60.92)	749 (63.74)	0.84 (0.76–0.93)	0.001
Admission for heart failure	88 (6.99)	108 (9.19)	0.72 (0.54–0.95)	0.020
Respiratory failure	148 (11.76)	126 (10.72)	1.06 (0.84–1.35)	0.623
ESRD requiring permanent dialysis	23 (2.54)	30 (3.62)	0.70 (0.41–1.20)	0.192

Values are given as number (%)

*HR* Hazard ratio, *SHR* Subdistribution hazard ratio, *CV* Cardiovascular, *PCI* Peripheral component interconnect, *CABG* Coronary artery bypass graft, *ESRD* End stage renal disease

**Fig. 3:**
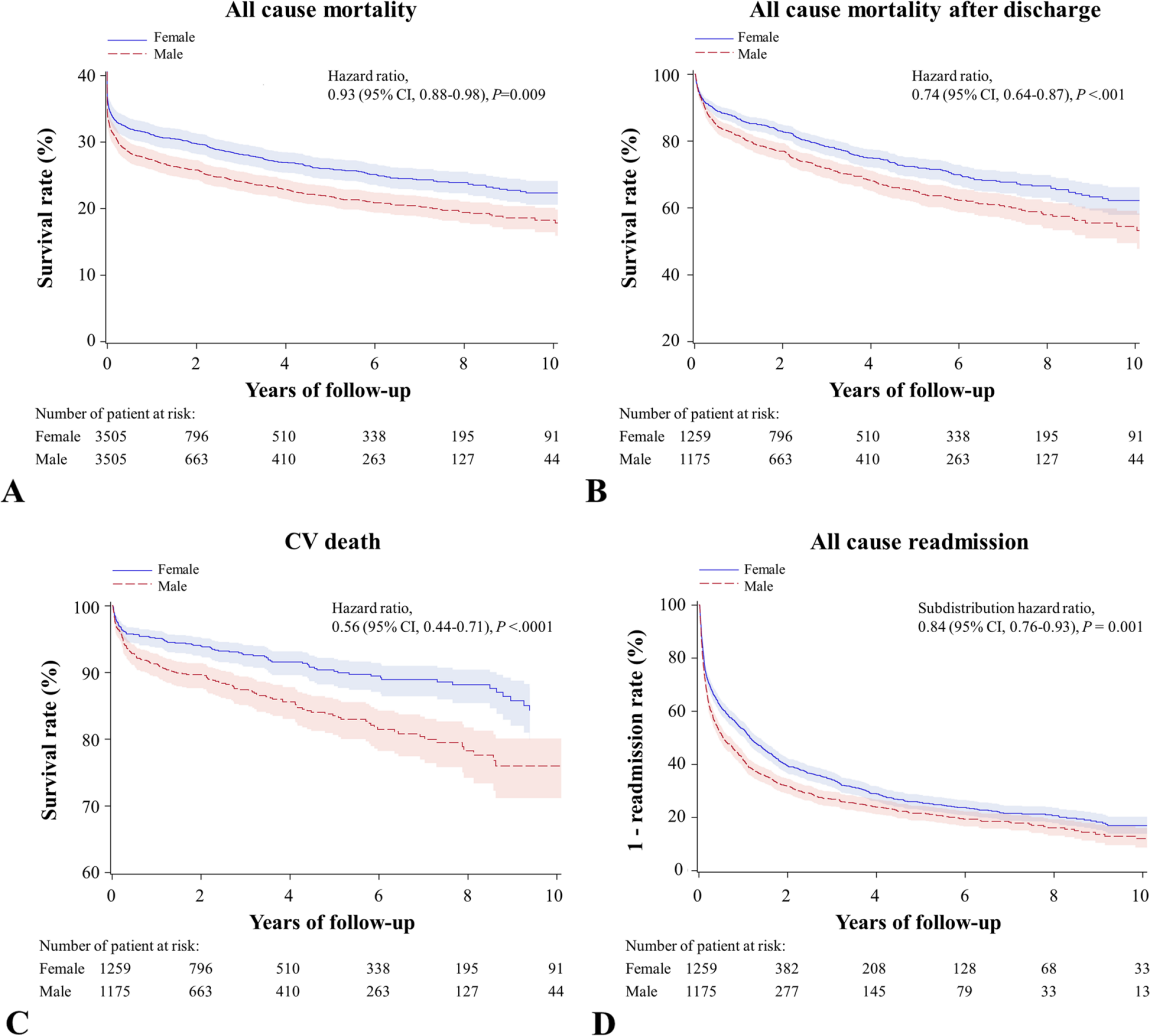
Kaplan–Meier survival curves for all-cause mortality (**A**), all-cause mortality after discharge (**B**), CV death (**C**), and all-cause readmission (**D**) of patients receiving ECMO support. CV, cardiovascular; CI, confidence interval; ECMO, extracorporeal membrane oxygenation support

Figure 3 summarizes the major primary outcomes and secondary outcomes of interest in this present study. Figure 3A and B illustrate the Kaplan–Meier survival curves for all-cause mortality and all-cause mortality after discharge, showing favorable outcomes in female patients undergoing ECMO support after more than a decade of follow-up. Furthermore, females also had a significantly lower risk of long-term cardiovascular death as compared with males (hazard ratio [HR], 0.56; 95% CI, 0.44–0.71; *P* < 0.0001; Fig. 3C). There was also a statistically significantly lower rate of readmission for any cause in the female group than in the male group (60.92% vs. 63.74%) during the follow-up period (subdistribution HR, 0.84; 95% CI, 0.76–0.93; *P* = 0.001; Fig. 3D).

### Subgroup analysis

Supplemental Tables 7 and 8 report the in-hospital and follow-up outcomes of subgroup analysis by different ECMO indications. The main outcomes of interest were similar to the entire cohort, revealing more favorable in female patients consistently in both of the cardiogenic-related and respiratory groups, including in-hospital mortality, all-cause mortality, and late cardiovascular death. Although these results were not statistically insignificant, it may be related to the lower sample size of the individual groups.

## Discussion

### Main findings

First, the use of ECMO has risen dramatically in Taiwan over the past decade, with a slightly decreased trend in the proportion of female patients. Second, we noted a trend of decreased in-hospital mortality in patients receiving ECMO support in Taiwan, among female, male, and overall patients. Third, female patients have lower risks of in-hospital mortality and ECMO-related complications as compared with males. Furthermore, females also have favorable long-term late outcomes such as all-cause mortality and readmission rate.

### Trends of ECMO use and outcome in Taiwan

The number of patients receiving ECMO support increased dramatically from 50 in 2001 to 1176 in 2017 in Taiwan, similar to the data given in the Extracorporeal Life Support Organization registry database [8]. In Taiwan, the NHI program reimburses almost all of the medical expenditures of ECMO treatment, and in response to the rising demands of ECMO treatment, the indications for reimbursement have been widely expanded since 2009 [12]. In addition to the economic issue, the reasons for the increase in ECMO use in Taiwan include the improvement of modern ECMO equipment, the increased familiarity surgeons with implantation techniques, and expanded indications. Moreover, the popularity of ECMO is also partially accountable, as not only medical centers but also many regional/district hospitals in Taiwan have the ability to implement ECMO treatment. These factors may also explain the amelioration of in-hospital mortality after ECMO support in Taiwan across the observed years. The overall in-hospital mortality rate was as high as 70% in 2001 but has decreased to about 60% in recent years, regardless of the sex of the patient. A recent multicenter observational study included 789 patients who underwent ECMO support from 2013 to 2018 and also revealed a comparable in-hospital mortality rate of 57.9% [13]. Peter et al. presented a retrospective cohort study using a population database from Germany, which indicated that ECMO patients had an overall 30-day in-hospital mortality rate of 61% [14]. Thus, the level of ECMO treatment and the trend in Taiwan are close to that of developed countries/regions.

### Sex differences in in-hospital outcomes

In the present study, we found that females have favored in-hospital mortality and lower risks of de novo dialysis and deep wound infection after ECMO. Wang et al. found a comparable adjusted in-hospital mortality rate (female: 56.7% vs. male: 60.2%; *P* = 0.59) of patients placed on ECMO treatment for cardiogenic shock [15]. Nevertheless, another study indicated that female sex was an independent predictor of in-hospital mortality [16]. In a single-center survey, female patients presented a higher risk of in-hospital mortality (HR, 2.86; 95% CI, 1.16–7.14; *P* = 0.02) [17], although that study included a small sample size (nine female patients). Because most of the population in documented articles had acute myocardial infarction–associated cardiogenic shock, the variable outcomes could be attributed to the sex differences in coronary artery disease. Females with acute myocardial infarction typically are older, present with worse conditions, and have a higher incidence of urgent/emergency coronary bypass surgeries than males do [18]. In addition, atypical presentation and delayed detection and referral to first aid also contributed to the unfavorable outcomes in female patients [19]. Females are also less like to suffer from a witnessed cardiac event and bystander cardiopulmonary resuscitation [20]. These factors may lead to the results of unfavored in-hospital mortality in female patients after ECMO in previous study and may be the confounding factors. The effect of covariates was thus mitigated with large sample size and meticulous matching in this study, revealing favorable in-hospital outcomes in females after ECMO support.

### Sex differences in late outcomes

There are only few documented articles investigating the outcomes of patients receiving ECMO support, and most discussed only short-term outcomes, such as in-hospital mortality or in-hospital complications, much less late outcomes during follow-up. Moreover, some articles included patients receiving overall mechanical circulatory support rather than focusing on ECMO patients [18, 19, 21, 22]. A single-center study analyzed ECMO patients after cardiotomy, finding comparable 30-day all-cause mortality between the sexes after PSM (56% male vs. 75% female; *P* = 0.262) [23]. Zhang et al. reported worse survival rates in females (11.1% vs. 53.1%, *P* = 0.05) that in males at up to 200 days of follow-up after the start of ECMO treatment [17]. As mentioned above, because of the very small sample size of these studies, their conclusions might be controversial. Our study is one of the limited studies in the literature demonstrating the sex effect on both early and late outcomes of ECMO patients on the basis of a national population database. In this study, females actually had more favorable late outcomes, especially all-cause mortality and cardiovascular death, than males did. Despite the advances in ECMO technology and popularity, females are still less likely to undergo ECMO treatment than males are. Males also receive more mechanical circulatory support other than ECMO, such as intra-aortic balloon pump, Impella, and ventricular assist devices [18, 19, 21]. Alasnag et al. also pointed out the perspective of underutilization in eligible female patients in their review article [19]. Therefore, we conclude it may be beneficial for females to receive ECMO support, and they should undergo treatment if available and necessary, based on the favorable late outcomes documented in this present study.

### Study strengths

To the best of our knowledge, this study is one of the first to analyze sex-related differences in outcomes of both in-hospital and long-term outcomes of patients receiving ECMO support. Previous articles mostly revealed only in-hospital outcomes with limited population inclusion and relative smaller sample sizes. Although ECMO treatment is indeed expensive, it is universally covered by the Taiwan NHI system, which reduces the influence of economic and social factors. Therefore, with the large-scale nationwide population and adequate statistical matching for analysis, our study provides sufficient information on short and late outcomes, including mortality and associated complications. This characteristic allows the present study to overcome the disadvantage of the small-population, single-institution study. With the relatively large sample size of this study, we adopted PSM to reduce the confounding of different variables and also to eliminate selection bias.

### Limitations

With the lack of code identification for extracorporeal cardiopulmonary resuscitation (ECPR) in NHIRD, we could not obtain the rate of ECPR in this study. The denominator of overall ECMO eligible patients across various indications could not be retrieved from the administrative database precisely. The detailed ECMO setting is not available from the Taiwan NHIRD, including the pump flow, cannula site and size, and mode. We identify veno-arterial (VA) and veno-venous (VV) ECMO by using ECMO indication as a proxy variable on the basis of current clinical practice [9, 24, 25]. The VA-ECMO is commonly used in patients with circulation failure and VV-ECMO is usually applied in patients with respiratory failure [1]. By carefully extracting the codes and indication stratification, the respiratory group of patients may represent most of the VV-ECMO population. In addition, because the NHIRD mainly stratifies cases by ICD codes and Taiwan NHI reimbursement codes, a small portion of cases may have been misclassified and/or may have had coding errors. Nevertheless, the Taiwan NHI system has strict principles regarding reimbursement indications. If cases with a deficiency of medical records or incorrect claims, high expenditure management would not be paid, making the precise payment to ECMO support. Therefore, this mechanism improved the accuracy of our data extraction. Several previous studies have validated the data in the NHIRD, as did the articles of our team, revealing good sensitivity, and specificity [24, 25].

## Conclusion

We conducted a nationwide cohort study to reveal sex differences in both short and late outcomes of patients undergoing ECMO support. Over the past two decades, the use of ECMO has increased dramatically in Taiwan, and there has been a trend of decreased in-hospital mortality, among female, male, and all patients overall. Female patients had favorable in-hospital and long-term survival outcomes, and the modern techniques and equipment used in ECMO have improved in the current era. Nevertheless, ECMO remains underutilized in eligible female patients. Therefore, based on the advances in clinical outcomes and underutilization of ECMO support in female patients, we conclude that females should undergo ECMO treatment if available and indicated.

## Supplementary Information

**Additional file 1: Supplementary Tables. Supplemental Table 1.** *ICD-9-CM *and *ICD-10-CM* codes used for diagnosis in the current study.** Supplemental Table 2.** Demographic, clinical, and surgical characteristics of the female and male groups.** Supplemental Table 3. **In-hospital mortality by age group.** Supplemental Table 4.** All-cause mortality during follow-up by age group.** Supplemental Table 5.** Mortality after discharge by age group.** Supplemental Table 6.** CV death after discharge by age group.** Supplemental Table 7.** In-hospital outcomes and ECMO-related complications in the female and male groups by ECMO indications.** Supplemental Table 8.** Long-term late outcomes in the female and male groups by ECMO indications.

## Data Availability

All data generated or analysed during this study are included in this published article and its supplementary information files.

## Abbreviations

ECMO: Extracorporeal membrane oxygenation
ICD-9-CM: *International Classi* *fi* *cation of Diseases, 9th Revision, Clinical Modi* *fi* *cation*
ICD-10-CM: *International Classi* *fi* *cation of Diseases,* *1* *0th Revision, Clinical Modi* *fi* *cation*
NHIRD: National Health Insurance Research Database
NHI: National Health Insurance
PSM: Propensity score matching
