# Supplementary material for: Sex differences in utilisation of extracorporeal membrane oxygenation support and outcomes in Taiwan
Source: BMC Anesthesiol. 2023 Mar 20;23:86. doi: 10.1186/s12871-023-02045-9 (PMC10026492; doi:10.1186/s12871-023-02045-9)
Supplement: Supplementary file 1 — Additional file 1: Supplementary Tables. Supplemental Table 1. ICD-9-CM and ICD-10-CM codes used for diagnosis in the current study. Supplemental Table 2. Demographic, clinical, and surgical characteristics of the female and male groups. Supplemental Table 3. In-hospital mortality by age group. Supplemental Table 4. All-cause mortality during follow-up by age group. Supplemental Table 5. Mortality after discharge by age group. Supplemental Table 6. CV death after discharge by age group. Supplemental Table 7. In-hospital outcomes and ECMO-related complications in the female and male groups by ECMO indications. Supplemental Table 8. Long-term late outcomes in the female and male groups by ECMO indications. [file 12871_2023_2045_MOESM1_ESM.docx]

| **Supplemental Table 1.** *ICD-9-CM* and *ICD-10-CM* codes used for diagnosis in the current study. | | | |
| --- | --- | --- | --- |
| Variable | ICD-9-CM code |  | ICD-10-CM code |
| **Comorbid conditions** |  |  |  |
| Hypertension | 401.xx–405.xx |  | I10-I15, N262 |
| Diabetes mellitus | 250.xx |  | E08-E13 |
| Heart failure | 428.xx |  | I50 |
| Prior myocardial infarction | 410.xx, 412.xx |  | I21-I22 |
| Peripheral arterial disease | 440.xx, 441.xx, 443.xx, 444.0x, 444.8x, 447.8x, 447.9x, 093.0, 437.3, 444.22, 447.1, 557.1, 557.9, V434 |  | I70, I71, I73, I75, I771, I790, I791, I792, I773, I779, I798, K551, K558, K559, Z958, Z959, I743, I744, I745, I748, I740, I7789 |
| Prior stroke | 430.xx–437.xx |  | I60-I62, I66, I65.1, I65.0, I65.8, I65.9, I63.6, I63.8, I63.9, G45.0, G45.8, G45.1, G45.2, G46.0, G46.1, G46.2, G45.9, G45.4, G46.3, G46.4, G46.5, G46.6, G46.7, G46.8, I67.0, I67.1, I67.2, I67.4, I67.5, I67.6, I67.7, I67.9, I68.0, I68.2, I68.8 |
| Chronic kidney disease | 580.xx–589.xx, 403.xx–404.xx, 016.0x, 095.4x, 236.9x, 250.4x, 274.1x, 442.1x, 447.3x, 440.1x, 572.4x, 642.1x, 646.2x, 753.1x, 283.11, 403.01, 404.02, 446.21 |  | A1811, D593, E102, E112, E132, I12, I13, K767, M103, M310, N00, N01, N02, N03, N04, N05, N06, N07, N08, N14, N150, N158, N159, N16, N171, N172, N18, N19, N200, N25, N261, N269, N27, Q61 |
| Liver cirrhosis | 571.5, 571.6, 571.2 |  | K702-K703, K741-K746 |
| Coagulopathy | 286.0-286.9, 287.1, 287.3-287.5, 289.81-289.82 |  | D65, D66, D67, D68, D69.1, D69.3, D69.4, D69.5, D69.6, D47.3 |
| COPD | 491.xx, 492.xx, 496.xx |  | J41-J44 |
| Depression | 296.2x, 296.3x, 296.5x, 296.82, 300.4x, 309.0, 309.1, 311, 648.4, 648.44, 780.79 |  | F32, F33 |
| **Complications** |  |  |  |
| Ischemic stroke | 433.xx–437.xx |  | I60-I62, I66, I65.1, I65.0, I65.8, I65.9, I63.6, I63.8, I63.9, G45.0, G45.8, G45.1, G45.2, G46.0, G46.1, G46.2, G45.9, G45.4, G46.3, G46.4, G46.5, G46.6, G46.7, G46.8, I67.0, I67.1, I67.2, I67.4, I67.5, I67.6, I67.7, I67.9, I68.0, I68.2, I68.8 |
| Hemorrhagic stroke | 430.xx–432.xx |  | I60-I62 |
| Deep wound infection | 998.3x, 998.5x, 038.xx, 790.7 |  | T81.30XA, T81.32XA, T81.31XA, T81.33XA, K68.11, A40, A41, R78.81 |

*ICD-9-CM, International Classiﬁcation of Diseases, Ninth Revision, Clinical Modiﬁcation;*

*ICD-10-CM, International Classiﬁcation of Diseases, Tenth Revision, Clinical Modiﬁcation.*

| **Supplemental Table 2.** Demographic, clinical, and surgical characteristics of the female and male groups. | | | | | | | | | |  |
| --- | --- | --- | --- | --- | --- | --- | --- | --- | --- | --- |
| Variable | Before matching | | |  | | After matching | | | |  |
|  | Female | Male | ASMD | |  | | Female | Male | ASMD | |
|  | (*n* = 3568) | (*n* = 8166) |  |  |  | | (*n* = 3505) | (*n* = 3505) |  |  |
| **Age (years)** | 56.75 ± 17.15 | 57.67 ± 15.16 | 0.06 | |  | | 56.66 ± 17.16 | 57.1 ± 15.07 | 0.03 | |
| **Monthly income, USD** |  |  | 0.09 | |  | |  |  | 0.04 | |
| 0–596 | 1003 (28.11) | 2370 (29.02) |  | |  | | 994 (28.36) | 1016 (28.99) |  | |
| 610–760 | 1295 (36.29) | 2630 (32.21) |  | |  | | 1270 (36.23) | 1182 (33.72) |  | |
| > 800 | 1246 (34.92) | 3088 (37.82) |  | |  | | 1241 (35.41) | 1307 (37.29) |  | |
| **Urbanization level** |  |  | 0.15 | |  | |  |  | 0.04 | |
| Urban | 12 (0.34) | 58 (0.71) |  | |  | | 0 (0) | 0 (0) |  | |
| Suburban | 2131 (59.73) | 4962 (60.76) |  | |  | | 2112 (60.26) | 2101 (59.94) |  | |
| Rural | 1122 (31.45) | 2417 (29.6) |  | |  | | 1108 (31.61) | 1102 (31.44) |  | |
| **Surgical year** |  |  | 0.05 | |  | |  |  | 0.02 | |
| 2000-2005 | 211 (5.91) | 416 (5.09) |  | |  | | 207 (5.91) | 195 (5.56) |  | |
| 2006-2009 | 656 (18.39) | 1461 (17.89) |  | |  | | 648 (18.49) | 621 (17.72) |  | |
| 2010-2013 | 1205 (33.77) | 2824 (34.58) |  | |  | | 1190 (33.95) | 1229 (35.06) |  | |
| 2014-2017 | 1496 (41.93) | 3465 (42.43) |  | |  | | 1460 (41.65) | 1460 (41.65) |  | |
| **Comorbid conditions** |  |  |  | |  | |  |  |  | |
| Hypertension | 1696 (47.53) | 4097 (50.17) | 0.05 | |  | | 1664 (47.48) | 1756 (50.1) | 0.05 | |
| Diabetes mellitus | 1129 (31.64) | 2664 (32.62) | 0.02 | |  | | 1102 (31.44) | 1136 (32.41) | 0.02 | |
| Heart failure | 704 (19.73) | 1482 (18.15) | 0.04 | |  | | 687 (19.6) | 650 (18.54) | 0.03 | |
| Prior myocardial infarction | 272 (7.62) | 1042 (12.76) | 0.17 | |  | | 270 (7.7) | 353 (10.07) | 0.08 | |
| Peripheral arterial disease | 416 (11.66) | 1076 (13.18) | 0.05 | |  | | 410 (11.7) | 448 (12.78) | 0.03 | |
| Prior stroke | 342 (9.59) | 784 (9.6) | 0.00 | |  | | 331 (9.44) | 329 (9.39) | 0.00 | |
| Chronic kidney disease | 1166 (32.68) | 3011 (36.87) | 0.09 | |  | | 1146 (32.7) | 1273 (36.32) | 0.08 | |
| Liver cirrhosis | 86 (2.41) | 233 (2.85) | 0.03 | |  | | 85 (2.43) | 92 (2.62) | 0.01 | |
| Coagulopathy | 141 (3.95) | 254 (3.11) | 0.05 | |  | | 133 (3.79) | 115 (3.28) | 0.03 | |
| COPD | 214 (6) | 808 (9.89) | 0.14 | |  | | 211 (6.02) | 270 (7.7) | 0.07 | |
| Depression | 216 (6.05) | 258 (3.16) | 0.14 | |  | | 194 (5.53) | 170 (4.85) | 0.03 | |
| Charlson’s Comorbidity Index Score | 2.5 ± 2.24 | 2.62 ± 2.24 | 0.05 | |  | | 2.5 ± 2.24 | 2.61 ± 2.26 | 0.05 | |
| **Hospital level** |  |  | 0.05 | |  | |  |  | 0.05 | |
| Medical center (teaching hospital) | 2429 (68.1) | 5358 (65.63) |  | |  | | 2389 (68.16) | 2305 (65.76) |  | |
| Regional / district hospital | 1138 (31.9) | 2806 (34.37) |  | |  | | 1116 (31.84) | 1200 (34.24) |  | |
| **Cumulative volume of ECMO between 2000 and 2017** |  |  | 0.09 | |  | |  |  | 0.08 | |
| 1st quartile (0-221) | 1007 (28.22) | 2558 (31.33) |  | |  | | 991 (28.27) | 1088 (31.04) |  | |
| 2nd quartile (222-601) | 996 (27.91) | 2235 (27.37) |  | |  | | 978 (27.9) | 973 (27.76) |  | |
| 3rd quartile (602-1,364) | 947 (26.54) | 1995 (24.43) |  | |  | | 934 (26.65) | 841 (23.99) |  | |
| 4th quartile (>1,365) | 618 (17.32) | 1378 (16.87) |  | |  | | 602 (17.18) | 603 (17.2) |  | |
| **Pre OP anti-HTN medication** |  |  |  | |  | |  |  |  | |
| ACEi/ ARB | 1224 (34.3) | 2966 (36.32) | 0.04 | |  | | 1200 (34.24) | 1258 (35.89) | 0.03 | |
| Beta blocker | 1079 (30.24) | 2391 (29.28) | 0.02 | |  | | 1057 (30.16) | 1037 (29.59) | 0.01 | |
| CCB | 846 (23.71) | 1867 (22.86) | 0.02 | |  | | 824 (23.51) | 814 (23.22) | 0.01 | |
| Alpha-blocker | 55 (1.54) | 308 (3.77) | 0.14 | |  | | 55 (1.57) | 75 (2.14) | 0.04 | |
| Thiazide | 119 (3.34) | 253 (3.1) | 0.01 | |  | | 116 (3.31) | 112 (3.2) | 0.01 | |
| Loop diuretics | 723 (20.26) | 1434 (17.56) | 0.07 | |  | | 701 (20) | 664 (18.94) | 0.03 | |
| Potassium-sparing diuretics | 284 (7.96) | 693 (8.49) | 0.02 | |  | | 276 (7.87) | 292 (8.33) | 0.02 | |
| Vasodilator | 727 (20.38) | 1907 (23.35) | 0.07 | |  | | 712 (20.31) | 755 (21.54) | 0.03 | |
| Nitrate | 538 (15.08) | 1482 (18.15) | 0.08 | |  | | 526 (15.01) | 568 (16.21) | 0.03 | |
| Number of anti-HTN drugs | 1.57 ± 1.75 | 1.63 ± 1.79 | 0.03 | |  | | 1.56 ± 1.75 | 1.59 ± 1.76 | 0.02 | |
| **Pre OP other medication** |  |  |  | |  | |  |  |  | |
| Statin | 628 (17.6) | 1587 (19.43) | 0.05 | |  | | 617 (17.6) | 659 (18.8) | 0.03 | |
| Antiplatelet | 668 (18.72) | 1976 (24.2) | 0.13 | |  | | 659 (18.8) | 770 (21.97) | 0.08 | |
| Warfarin | 244 (6.84) | 370 (4.53) | 0.10 | |  | | 234 (6.68) | 177 (5.05) | 0.07 | |
| NOACs | 5 (0.14) | 19 (0.23) | 0.02 | |  | | 5 (0.14) | 8 (0.23) | 0.02 | |
| OHA | 661 (18.53) | 1630 (19.96) | 0.04 | |  | | 651 (18.57) | 696 (19.86) | 0.03 | |
| Insulin | 267 (7.48) | 504 (6.17) | 0.05 | |  | | 255 (7.28) | 246 (7.02) | 0.01 | |
| Digoxin | 207 (5.8) | 437 (5.35) | 0.02 | |  | | 198 (5.65) | 195 (5.56) | 0.00 | |
| **ECMO indication** |  |  | 0.23 | |  | |  |  | 0.00 | |
| Postcardiotomy | 1548 (43.39) | 3574 (43.77) |  | |  | | 1525 (43.51) | 1525 (43.51) |  | |
| Cardiogenic shock, myocarditis or AMI | 825 (23.12) | 2246 (27.5) |  | |  | | 811 (23.14) | 811 (23.14) |  | |
| Respiratory | 791 (22.17) | 1602 (19.62) |  | |  | | 780 (22.25) | 780 (22.25) |  | |
| Trauma | 120 (3.36) | 335 (4.1) |  | |  | | 111 (3.17) | 111 (3.17) |  | |
| Others | 284 (7.96) | 409 (5.01) |  | |  | | 278 (7.93) | 278 (7.93) |  | |
| **ECMO duration** | 5.37 ± 5.05 | 5.4 ± 6.75 | 0.01 | |  | | 5.36 ± 5.03 | 5.46 ± 5.78 | 0.02 | |
| Values are given as number (%) or mean ± standard deviation. ASMD, absolute standardized mean difference; USD, US dollar; ECMO, Extracorporeal membrane oxygenation; OP, operation; COPD, chronic obstructive pulmonary disease; AMI, acute myocardial infarction. | | | | | | | | | |  |

**Supplemental Table 3.** In-hospital mortality by age group.

| Age group | Overall | Female | Male | P for chi-square  (Sex disparity) |
| --- | --- | --- | --- | --- |
| 20-29 | 52.47 | 46.13 | 57.14 | 0.005 |
| 30-39 | 53.32 | 48.54 | 56.46 | 0.007 |
| 40-49 | 60.51 | 59.69 | 60.79 | 0.680 |
| 50-59 | 65.65 | 68.21 | 64.87 | 0.111 |
| 60-69 | 70.85 | 71.05 | 70.77 | 0.880 |
| 70-79 | 77.69 | 75.35 | 78.88 | 0.064 |
| ≧80 | 83.94 | 82.86 | 84.43 | 0.552 |
| P for linear trend | <0.001 | <0.001 | <0.001 |  |

Data presented as percentage (%).

**Supplemental Table 4.** All-cause mortality during follow-up by age group.

| Age group | Overall | Female | Male | P for chi-square  (Sex disparity) |
| --- | --- | --- | --- | --- |
| 20-29 | 58.30 | 51.76 | 63.12 | 0.003 |
| 30-39 | 60.61 | 54.18 | 64.84 | <0.001 |
| 40-49 | 69.92 | 67.71 | 70.67 | 0.237 |
| 50-59 | 75.46 | 77.31 | 74.89 | 0.201 |
| 60-69 | 81.00 | 80.51 | 81.21 | 0.661 |
| 70-79 | 88.49 | 85.79 | 89.87 | 0.005 |
| ≧80 | 92.91 | 92.50 | 93.10 | 0.746 |
| P for linear trend | <0.001 | <0.001 | <0.001 |  |

Data presented as percentage (%).

**Supplemental Table 5.** Mortality after discharge by age group.

| Age group | Overall | Female | Male | P for chi-square  (Sex disparity) |
| --- | --- | --- | --- | --- |
| 20-29 | 12.26 | 10.46 | 13.94 | 0.344 |
| 30-39 | 15.63 | 10.98 | 19.24 | 0.007 |
| 40-49 | 23.82 | 19.89 | 25.19 | 0.150 |
| 50-59 | 28.54 | 28.64 | 28.52 | 0.972 |
| 60-69 | 34.80 | 32.67 | 35.70 | 0.398 |
| 70-79 | 48.42 | 42.37 | 52.01 | 0.042 |
| ≧80 | 55.86 | 56.25 | 55.67 | 0.947 |
| P for linear trend | <0.001 | <0.001 | <0.001 |  |

Data presented as percentage (%).

**Supplemental Table 6.** CV death after discharge by age group.

| Age group | Overall | Female | Male | P for chi-square  (Sex disparity) |
| --- | --- | --- | --- | --- |
| 20-29 | 4.09 | 2.61 | 5.45 | 0.201 |
| 30-39 | 5.86 | 3.25 | 7.89 | 0.020 |
| 40-49 | 11.62 | 8.29 | 12.79 | 0.104 |
| 50-59 | 11.21 | 9.39 | 11.72 | 0.341 |
| 60-69 | 12.95 | 10.36 | 14.04 | 0.145 |
| 70-79 | 18.53 | 13.56 | 21.48 | 0.032 |
| ≧80 | 22.76 | 16.67 | 25.77 | 0.218 |
| P for linear trend | <0.001 | <0.001 | <0.001 |  |

Data presented as percentage (%). CV, cardiovascular.

| **Supplemental Table 7.** In-hospital outcomes and ECMO-related complications in the female and male groups by ECMO indications. | | | | | | | | |
| --- | --- | --- | --- | --- | --- | --- | --- | --- |
| Variable | Postcardiotomy | | Cardiogenic shock, myocarditis or AMI | | Respiratory | | Trauma | |
|  | OR/B  (95% CI) | *P*  *value* | OR/B  (95% CI) | *P*  *value* | OR/B  (95% CI) | *P*  *value* | OR/B  (95% CI) | *P*  *value* |
| In-hospital mortality | 1.01 (0.87-1.18) | 0.906 | 0.99 (0.80-1.22) | 0.914 | 1.00 (0.81-1.23) | 1.000 | 0.92 (0.52-1.62) | 0.773 |
| New onset stroke | 0.81 (0.59-1.10) | 0.181 | 1.72 (1.00-2.93) | 0.049 | 1.13 (0.65-1.95) | 0.675 | 0.66 (0.11-4.03) | 0.653 |
| New onset ischemic stroke | 0.70 (0.49-0.99) | 0.044 | 1.21 (0.60-2.41) | 0.598 | 1.20 (0.52-2.80) | 0.668 | 0.66 (0.11-4.03) | 0.653 |
| New onset hemorrhagic stroke | 1.36 (0.76-2.43) | 0.305 | 3.35 (1.43-7.86) | 0.005 | 1.18 (0.61-2.27) | 0.618 | - | - |
| Fasciotomy or amputation | 1.27 (0.76-2.11) | 0.366 | 0.50 (0.17-1.46) | 0.204 | 0.80 (0.31-2.03) | 0.636 | 1.26 (0.33-4.83) | 0.734 |
| Prolonged ventilation (>=7 days) | 0.83 (0.71-0.96) | 0.011 | 0.93 (0.76-1.14) | 0.475 | 1.03 (0.82-1.28) | 0.822 | 0.56 (0.33-0.95) | 0.032 |
| Newly-onset dialysis | 0.95 (0.82-1.10) | 0.524 | 0.78 (0.64-0.95) | 0.015 | 0.85 (0.69-1.03) | 0.101 | 0.64 (0.38-1.10) | 0.105 |
| post-ECMO deep wound infection | 1.01 (0.82-1.24) | 0.958 | 1.33 (1.01-1.77) | 0.046 | 0.81 (0.66-0.99) | 0.039 | 0.69 (0.29-1.62) | 0.389 |
| PRBC amount (U) | -0.09 (-1.04-0.86) | 0.847 | 0.84 (0.11-1.56) | 0.024 | -0.05 (-0.92-0.82) | 0.910 | -1.65 (-4.68-1.39) | 0.287 |
| FFP amount (U) | -0.73 (-1.73-0.27) | 0.155 | 0.70 (-0.40-1.80) | 0.212 | 0.65 (-0.66-1.95) | 0.330 | 2.24 (-1.63-6.11) | 0.255 |
| Platelet amount (U) | 0.04 (-0.86-0.95) | 0.924 | 1.98 (0.81-3.15) | 0.001 | -1.73 (-2.96--0.50) | 0.006 | -1.01 (-5.5-3.49) | 0.659 |
| Ventilator (days) | -1.17 (-1.92--0.43) | 0.002 | -0.26 (-1.08-0.55) | 0.529 | -0.16 (-1.28-0.97) | 0.786 | -3.15 (-5.81--0.49) | 0.021 |
| ICU duration (days) | -0.84 (-1.56--0.11) | 0.024 | -0.06 (-0.92-0.80) | 0.889 | -0.02 (-1.07-1.02) | 0.964 | -2.22 (-4.81-0.37) | 0.092 |
| Hospital stays (days) | -4.01 (-6.59--1.42) | 0.002 | 0.42 (-2.26-3.10) | 0.759 | 2.22 (-1.32-5.75) | 0.218 | -2.27 (-9.24-4.70) | 0.522 |
| In hospital cost (USD x 10^3^) | -4.60 (-6.55--2.66) | <0.001 | 0.16 (-1.68-2.00) | 0.864 | 1.21 (-1.17-3.58) | 0.320 | -7.03 (-16.04-1.98) | 0.126 |
| ECMO, Extracorporeal membrane oxygenation; AMI, acute myocardial infarction; OR, odds ratio; B, unstandardized coefficient; PRBC, packed red blood cell; FFP, fresh frozen plasma; ICU, intensive care unit; USD, United States dollar. | | | | | | | | |

| **Supplemental Table 8.** Long-term late outcomes in the female and male groups by ECMO indications. | | | | | | | | |  |  |
| --- | --- | --- | --- | --- | --- | --- | --- | --- | --- | --- |
| Variable | Postcardiotomy | | Cardiogenic shock, myocarditis or AMI | | Respiratory | | Trauma | |  |  |
|  | HR or SHR | *P*  *value* | HR or SHR | *P*  *value* | HR or SHR | *P*  *value* | HR or SHR | *P*  *value* |  |  |
|  | (95% CI) |  | (95% CI) |  | (95% CI) |  | (95% CI) |  |  |  |
| All-cause mortality | 0.93 (0.86-1.01) | 0.092 | 0.98 (0.88-1.09) | 0.717 | 0.94 (0.84-1.06) | 0.296 | 0.94 (0.69-1.29) | 0.719 |  |  |
| CV death | 0.49 (0.36-0.68) | <.0001 | 0.76 (0.49-1.18) | 0.220 | 0.51 (0.17-1.52) | 0.228 | - | - |  |  |
| Revascularization (PCI or CABG) | 0.37 (0.14-0.94) | 0.037 | 0.11 (0.01-0.83) | 0.033 | - | - | - | - |  |  |
| Acute myocardial infarction | 0.79 (0.38-1.63) | 0.526 | 0.32 (0.13-0.8) | 0.015 | - | - | - | - |  |  |
| Ischemic stroke | 0.71 (0.37-1.38) | 0.312 | 0.30 (0.06-1.43) | 0.130 | - | - | - | - |  |  |
| Hemorrhagic stroke | 0.67 (0.27-1.66) | 0.386 | 0.90 (0.13-6.36) | 0.916 | - | - | - | - |  |  |
| All-cause of readmission | 0.82 (0.71-0.95) | 0.007 | 0.70 (0.56-0.87) | 0.002 | 1.05 (0.85-1.31) | 0.642 | 0.90 (0.51-1.57) | 0.697 |  |  |
| Admission for HF | 0.75 (0.52-1.06) | 0.106 | 0.66 (0.38-1.13) | 0.130 | 0.93 (0.35-2.49) | 0.891 | - | - |  |  |
| Respiratory failure | 1.07 (0.75-1.52) | 0.707 | 0.98 (0.58-1.66) | 0.945 | 1.42 (0.9-2.24) | 0.129 | - | - |  |  |
| ESRD requiring permanent dialysis | 0.44 (0.11-1.67) | 0.226 | 0.91 (0.43-1.94) | 0.805 | 0.75 (0.3-1.91) | 0.546 | - | - |  |  |
| AMI, acute myocardial infarction; HR, hazard ratio; SHR, subdistribution hazard ratio; CV, cardiovascular; HF, heart failure; ESRD, end stage renal disease | | | | | | | | | |  |
